# The Mouth a Human Culture Tube

**Published:** 1898-07

**Authors:** F. B. Rees


### The Mouth a Human Culture Tube.

BY F. B. REES, D.D.S.

Read before the Cincinnati Academy of Dentistry.

It is concerning the reflex disturbances and indirect injuries brought about by diseased teeth and an uncleanly mouth that this paper will treat.

It is a fact that whatever tends to create discomfort to the individual will do injury to either mental or bodily health. This statement does not mean that securing comfort in every sense of that word is the end and object of human desires. It is to comfort in the sense of freedom from pain and disturbed functions that we here limit the meaning. It is well known that we are conscious of the existence of scarcely any organ of the body when it is in a normal condition. It is said, the man who knows he has a stomach has a diseased one.

It is an established fact that the ordinary decay of the teeth is the result of the action of microbes. The almost uniform temperature of the mouth makes it a most efficient type of culture oven; its fluids are rich in nutritious substances; the abundant supply of oxygen gives opportunity for the production of oxidized organic products, and hence acid-forming fermentations are constantly occurring. The acids attack the enamel and thus expose the real organic living tissues to injury. Even in a mouth full of sound teeth a great variety of microbes may be found, and if the teeth be not regularly cleansed these microbes will get mixed up with the food and be swallowed, thus introducing them into the stomach and intestines. A not uncommon affection is a chronic suppuration in pyorrhea alveolaris, which will naturally introduce pus-forming microbes constantly into the stomach.

It is very clear that both the number and variety of the bacteria of the mouth are constantly being augmented by new germs that enter with the air, food and drink. In the course of a few years hundreds of kinds of bacteria would therefore become established in the mouth if the majority of them did not perish, sooner or later, in the struggle for existence. Constantly new varieties are appearing and disappearing. Almost all have the peculiarity that they will not grow in any of the usual culture media.

It is said that a human being has two minds—the conscious and the unconscious.

The conscious mind is the animating principle of the cerebrospinal system of nerves, and the unconscious mind, in a like manner, vitalizes the sympathetic nervous system. When alarming reports come from the outer world (such as a sudden compression of an exposed nerve or an inflamed tissue) to the cerebro-spinal centers and the harmony of its domain is seriously disturbed, they express their discomfort in the language of pain, while the alarm cry of the sympathetic system is told in the form of functional derangements. By repeated inter-communications the two nervous systems can be mutually cognizant of each other's operations, and aid or antagonize each other in their daily work, as they are harmonious or discordant.

With the mouth in a suppurative or inflamed condition the process of mastication will be slighted to avoid pain; and here is where the sympathetic system will rebel and allow the various organs to become deranged. The defense being broken down the battle goes merrily on until some conscientious dentist steps in and removes the cause, when, presto, a great many distressing diseases vanish.

I don't know of any better way to make this clear than to describe one case in particular that came under my notice for treatment:

Patient of forty years was having her eyes and stomach treated, and thought while thus engaged she might as well have her mouth looked after. The germs in the "human culture tube" were being recruited so fast that there was a regular battle going on to see which would go to the front first and fight the common enemy—health. The six anterior inferior teeth were good, with the exception of a few small cavities. The bicuspids and molars were off even with the gum, and in some cases below the gum. It was simply a mass of inflamed gum, broken roots, and pus. I removed all the roots, and after a few days made her a temporary plate so she could masticate her food properly. She returned home and in about two months wrote me that she was enjoying better health than she had for years—eyes were all right and stomach gave her no trouble. I believe that, without putting her mouth in a proper hygienic state, she would have continued to treat her various ailments until the end of her life.

As a recapitulation of the foregoing, we are led to the conclusion that no point of personal hygiene is of more importance than taking proper care of the mouth and teeth. The mouth is the very portal of the human body. If children's teeth and the condition of their mouths were conscientiously cared for from childhood on, much suffering and ill-health might be prevented. Under the strained and artificial conditions of modern life the tooth brush is not alone sufficient for the care of the teeth, but antiseptic washes should be regularly used, especially before retiring. If we could impress this fact on parents our work would be made a great deal more pleasant, and just as profitable.

DISCUSSION.

Dr. W. T. McLean: The tripod of endeavor by scientific men is indeed *comfort* and *freedom* from pain, and, we might add, a *competence.* I cannot take issue with the essayist upon the fact of the mouth being a culture tube, for in my short experience I have seen some mouths that would simulate a volcanic eruption of putrescent material. The variety of these vegetable germs is countless, and I believe some are necessary for comfort, while others are indeed quite injurious. I coincide fully regarding the system's lesions spoken of. I am aware of my services rendering the patient treated untold benefit from treating many conditions, as Dr. Rees has mentioned. In speaking of the *motor, sensory* and *sympathetic* nervous system, the patient to be enjoying physical comfort, those various divisions of the nervous system must be in perfect harmony; if they are not, disturbance in the organism occurs, and inconvenience or pain is the result. Regarding the practical case cited, I have had many similar where the reflex conditions seemed so far distant from the seat of the lesion that the regular practitioner would seem prone to believe I was joking when in consultation. I offered suggestions similar to that which the essayist has illustrated. There is no question but the mouth must be in a normal condition before any reflex or systemic malady can be successfully treated. The hygiene of the mouth should be fully explained to every patient and each mother should be taught how to cleanse and care for her children's teeth, and children should be taught to visit a dentist for examination and treatment at as early a age as possible. I congratulate the essayist on his scientific opinions, and the masterly way he has treated this subject.

